# MCT4 surpasses the prognostic relevance of the ancillary protein CD147 in clear cell renal cell carcinoma

**DOI:** 10.18632/oncotarget.5593

**Published:** 2015-09-10

**Authors:** Pascale Fisel, Viktoria Stühler, Jens Bedke, Stefan Winter, Steffen Rausch, Jörg Hennenlotter, Anne T. Nies, Arnulf Stenzl, Marcus Scharpf, Falko Fend, Stephan Kruck, Matthias Schwab, Elke Schaeffeler

**Affiliations:** ^1^ Dr. Margarete Fischer-Bosch Institute of Clinical Pharmacology, Stuttgart, Germany; ^2^ University of Tuebingen, Tuebingen, Germany; ^3^ Department of Urology, University Hospital Tuebingen, Tuebingen, Germany; ^4^ German Cancer Consortium (DKTK) and German Cancer Reseach Center (DKFZ), Heidelberg, Germany; ^5^ Institute of Pathology and Neuropathology, University Hospital Tuebingen, Tuebingen, Germany; ^6^ Department of Clinical Pharmacology, University Hospital Tuebingen, Tuebingen, Germany

**Keywords:** CD147, MCT4, DNA methylation, ccRCC, prognosis, Pathology Section

## Abstract

Cluster of differentiation 147 (CD147/*BSG*) is a transmembrane glycoprotein mediating oncogenic processes partly through its role as binding partner for monocarboxylate transporter MCT4/*SLC16A3*. As demonstrated for MCT4, CD147 is proposed to be associated with progression in clear cell renal cell carcinoma (ccRCC). In this study, we evaluated the prognostic relevance of CD147 in comparison to MCT4/*SLC16A3* expression and DNA methylation.

**Methods:**

CD147 protein expression was assessed in two independent ccRCC-cohorts (*n* = 186, *n* = 59) by immunohistochemical staining of tissue microarrays and subsequent manual as well as automated software-supported scoring (Tissue Studio, Definien sAG). Epigenetic regulation of CD147 was investigated using RNAseq and DNA methylation data of The Cancer Genome Atlas. These results were validated in our cohort. Relevance of prognostic models for cancer-specific survival, comprising CD147 and MCT4 expression or *SLC16A3* DNA methylation, was compared using chi-square statistics.

**Results:**

CD147 protein expression generated with Tissue Studio correlated significantly with those from manual scoring (*P* < 0.0001, r_S_ = 0.85), indicating feasibility of software-based evaluation exemplarily for the membrane protein CD147 in ccRCC. Association of CD147 expression with patient outcome differed between cohorts. DNA methylation in the CD147/*BSG* promoter was not associated with expression. Comparison of prognostic relevance of CD147/*BSG* and MCT4/*SLC16A3*, showed higher significance for MCT4 expression and superior prognostic power for DNA methylation at specific CpG-sites in the *SLC16A3* promoter (e.g. CD147 protein: *P* = 0.7780, Harrell's c-index = 53.7% *vs*. DNA methylation: *P* = 0.0076, Harrell's c-index = 80.0%).

**Conclusions:**

Prognostic significance of CD147 protein expression could not surpass that of MCT4, especially of *SLC16A3* DNA methylation, corroborating the role of MCT4 as prognostic biomarker for ccRCC.

## INTRODUCTION

Cluster of differentiation 147 (CD147), also known as extracellular matrix metalloproteinase inducer (EMMPRIN) or basigin, encoded by the *BSG* gene, is a transmembrane glycoprotein, which is involved in various physiological as well as pathophysiological processes. In many solid tumors CD147 is overexpressed and associated with tumor progression, invasion and metastasis [1]. The oncogenic potential is attributed in parts to its well-known function to induce matrix metalloproteinases (MMPs) by tumor cells and mainly by neighboring stromal fibroblasts [2, 3]. In addition, CD147 has been shown to increase the production of vascular endothelial growth factor (VEGF), thereby promoting tumor angiogenesis [4, 5]. Furthermore, CD147 represents the obligatory binding partner for several proteins involved in carcinogenesis, e.g., the monocarboxylate transporters (MCT) 1 and MCT4 (encoded by *SLC16A1* and *SLC16A3*, respectively) [6], which mediate the export of lactate from highly glycolytic tumor cells. Inhibition of MCT1 and MCT4, thereby interfering with the glycolytic metabolism of tumor cells, is an attractive approach in cancer therapy. In clear cell renal cell carcinoma (ccRCC), which is characterized by a glycolytic Warburg phenotype, MCT4 has not only been shown to be a metabolic target to reverse the Warburg effect [7], as shown by Gerlinger et al in a genome-wide siRNA screening study in RCC cell lines, but is also proposed to be a prognostic marker for patient outcome. In a recent study, we could show that MCT4 expression is regulated by DNA methylation in the *SLC16A3* promoter and that DNA methylation status at single cytosine phosphate guanine (CpG) sites is predictive for patient survival [8]. Due to its tumorigenic properties, CD147 also represents a promising target for therapeutic intervention [9, 10] and it is suggested that CD147 expression alone or together with other factors such as VEGF expression, could also serve as a marker for prognosis and outcome in ccRCC [11–13].

The most widely applied method for the identification and evaluation of prognostic biomarkers is immunohistochemistry, although this approach does not allow absolute quantification of protein expression. Manual evaluation of immunoreactivity requires experienced pathologists and remains a subjective approach influenced by intra- and inter-observer variability. To overcome these limitations, the use of the automated image analysis software Tissue Studio v.3.6 (Definiens AG, Munich, Germany) might represent an attractive alternative to conventional visual evaluation, enabling more objective, reproducible, and precise quantification of biomarker expression. The use of Tissue Studio for the quantification of immunoreactivity has been evaluated for selected markers expressed in the cytoplasm, the nucleus or in both compartments in prostate cancer tissue [14]. However, the present study is the first to elucidate the applicability of this software for membranous biomarker staining in ccRCC tissue.

This study aimed to elucidate and validate the associations of CD147 expression with ccRCC tumor progression and patient outcome (cancer-specific survival). Therefore, the utility and feasibility of the automated image analysis software Tissue Studio v.3.6 for semi-quantitative evaluation of CD147 protein expression in ccRCC tissue in comparison to manual semi-quantitative scoring was assessed. The prognostic relevance of CD147 expression was compared to that of MCT4 expression and *SLC16A3* promoter DNA methylation in different cohorts. In addition, the prognostic ability of different models consisting of combinations of the potentially prognostic variables was assessed to identify the most qualified prognostic factor for patient outcome in ccRCC.

## RESULTS

### Patient cohorts

Patient characteristics of the three investigated cohorts are given in Table 1 and are described in detail in Supplementary Material.

**Table 1:** Detailed patient characteristics of the 3 investigated ccRCC cohorts

		Cohort 1		Cohort 2 (TCGA)		Cohort 3	
Variable		n	%	n	%	n	%
**no. of patients**		186		530		59	
**sex**	m	131	70.4	342	64.5	34	57.6
	f	55	29.6	188	35.5	25	42.4
**age^a^ [years]** median (range)		64	17-90	61	26-90	64	35-87
**tumor size [cm]** median (range)		4.4	0.3-12.5	-	-	5.5	1.4-16
**T**	1	129	69.4	270	50.9	34	57.6
	2	0	0	69	13	6	10.2
	3	57	30.6	180	34	19	32.2
	4	-	-	11	2.1	-	-
**N**	0	183	98.4	238	44.9	52	88.1
	1	3	1.6	17	3.2	7	11.9
	x	-	-	275	51.9	-	-
**M**	0	164	88.2	423	79.8^b^	50	84.7
	1	22	11.8	79	14.9^b^	9	15.3
	x	-	-	26	4.9^b^	-	-
**G**	1	31	16.7	13	2.5^b^	13	22
	2	133	71.5	228	43^b^	38	64.4
	3/4	22	11.8	281	53^b^	8	13.6
	x	-	-	5	0.9^b^	-	-
**L**	0	180	96.8	-	-	56	94.9
	1	6	3.2	-	-	3	5.1
**V**	0	144	77.4^c^	-	-	44	74.6
	1	41	22^c^	-	-	14	23.7
	2	1	0.5^c^	-	-	1	1.7
**R**	0	174	93.5^b^	-	-	55	93.2
	1	11	5.9^b^	-	-	4	6.8
**tumor necrosis**							
no		118	63.4	269	50.8^b^	51	86.4
yes		68	36.6	230	43.4^b^	8	13.6
**sarcomatoid differentiation**							
no		178	95.7	-	-	55	93.2
yes		8	4.3			4	6.8
**follow-up time [years]**		8	0-19.2	3.01	0-10.4	4.5	0-7.3
**recurrence**							
no		142	76.3	-	-	47	79.7
yes		44	23.7	-	-	12	20.3
**overall survival**							
alive		119	64	363	68.5^b^	49	83.1
deceased		67	36	153	28.9^b^	10	16.9
**cancer-specific survival**							
alive/non-cancer-related death		156	83.9	411	77.5^b^	51	86.4
cancer-related death		30	16.1	100	18.9^b^	8	13.6

Abbreviations: T: primary tumor; N: regional lymph nodes; M: distant metastasis; G: grading; L: invasion into lymph vessels; V: invasion into veins; R: resection status.

a Age at surgery for cohort 1 and 3, age at initial pathologic diagnosis for cohort 2 (TCGA).

b Percentages do not sum up to 100% because of missing values.

c Percentages do not sum up to 100% due to rounding.

### Evaluation of CD147 protein expression

**Figure 1:**
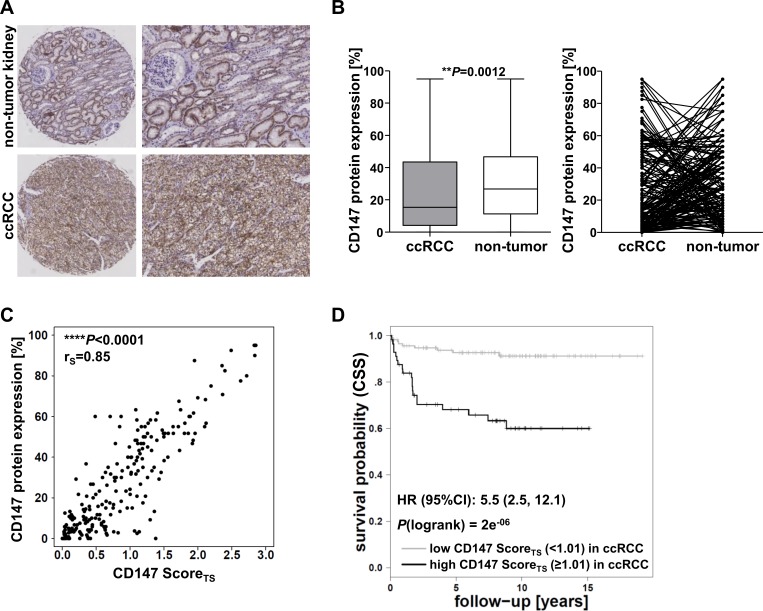
Evaluation of CD147 protein expression in cohort 1 **A.** Representative IHC staining of CD147 in ccRCC and non-tumor tissue. In non-tumor tissue CD147 protein is expressed in the basolateral membrane of proximal tubule cells, ccRCC tissue shows circumferential membranous CD147 protein expression. **B.** Evaluation of CD147 protein expression levels derived from the semi-quantitative scoring system and comparison between corresponding ccRCC and non-tumor tissue samples. **C.** Correlation analysis of the manually assigned semi-quantitative CD147 protein expression score (CD147 protein expression [%]) and the software-derived score from Tissue Studio (CD147 Score_TS_). **D.** Kaplan-Meier analysis of ccRCC patient survival (endpoint: cancer-specific survival, CSS) based on CD147 protein expression levels in ccRCC tissue of cohort 1; cutoffs were determined by conditional inference tree models.

CD147 protein expression was investigated by immunohistochemical staining of TMAs in the ccRCC cohort 1. The specificity of the CD147 antibody used was validated in siRNA knockdowns in four RCC cell lines (Supplementary Figure S1). Figure 1A shows representative CD147 staining of non-tumor and ccRCC tissue. In order to determine protein expression in ccRCC and adjacent non-tumor tissue, a semi-quantitative scoring system, derived from staining intensity and the percentage of stained cells, was assigned (see Materials and Methods) by two independent investigators. CD147 was expressed in ccRCC as well as in non-tumor tissue with high interindividual variability. Overall, the expression was higher in non-tumor tissue compared to ccRCC tissue in 66% of the cases of matching non-tumor and ccRCC tissues; in 34% of the cases the expression was higher in ccRCC tissue (Figure 1B).

CD147 protein expression in ccRCC tissue was additionally evaluated by use of the image analysis software Tissue Studio v.3.6 (Definiens AG, Munich, Germany), which allows automated detection of user-defined regions of interest in tissue cores and subsequent evaluation of staining intensities in specific regions of interest, i.e. solely in tumor regions. The method was adapted for the quantification of CD147 staining in ccRCC tissue. A detailed description is given in Materials and Methods.

The accuracy of the protein expression score derived from Tissue Studio (CD147 Score_TS_) was verified by correlation analyses with the manually assigned semi-quantitative score (CD147 protein expression [%]). As shown in Figure 1C, the applied scores for CD147 protein expression of both methods correlated significantly in ccRCC tissue of the two investigated cohorts (r_S_ = 0.85; *P* < 0.0001). Therefore, the score derived from Tissue Studio (CD147 Score_TS_) was used for further evaluations.

### CD147 protein expression in cohort 1

The evaluation of CD147 protein expression in ccRCC tissue of the cohort 1 by Tissue Studio revealed an increased protein expression along with higher tumor stages (*P* = 1.65e^−10^) and the occurrence of metastases (*P* = 1.44e^−03^). Kaplan-Meier analyses showed a relation between CD147 expression and the probability of cancer-specific survival, in that high expression levels were associated with poor survival based on a cutoff determined by conditional inference tree models (Hazard Ratio (HR) = 5.5, 95% Confidence Interval (CI) (2.5, 12.1); *P*(logrank) = 2e^−06^) (Figure 1D).

### CD147 mRNA levels and DNA methylation in cohort 2

**Figure 2:**
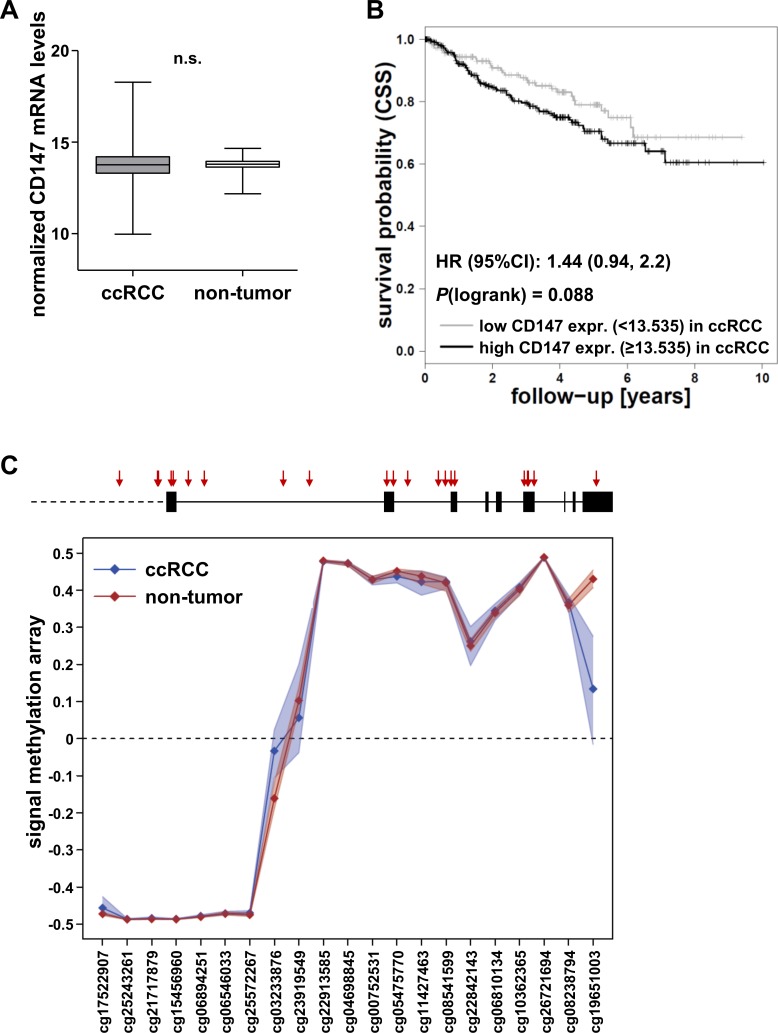
Evaluation of CD147 mRNA levels and CD147/*BSG* DNA methylation in ccRCC cohort 2 (TCGA) **A.** CD147 mRNA levels in the ccRCC cohort 2 (TCGA) in tumor (*n* = 529) and non-tumor tissue (*n* = 72). **B.** Kaplan-Meier curves of cancer-specific survival (CSS, defined according to [19]) for patients with high and low CD147 mRNA levels with cutoffs determined by conditional inference tree models. **C.** Scheme of the *BSG* gene locus with the investigated CpG sites (red arrows); DNA methylation in the *BSG* gene region in ccRCC (blue) and non-tumor tissue (red) is not considerably different.

In addition, RNAseq data of the ccRCC cohort 2 from The Cancer Genome Atlas (TCGA; *n* = 530) was analysed. Again, CD147 mRNA expression was observed in non-tumor as well as in ccRCC tissue at comparable levels, though the variability was higher in ccRCC tissue (Figure 2A). However, mRNA levels in ccRCC tissue were not associated with patient survival (Figure 2B; HR = 1.44, 95%CI (0.94, 2.2); *P*(logrank) = 0.088).

As previously shown for MCT4/*SLC16A3*, gene expression might be influenced by aberrant DNA methylation. Based on this hypothesis, we examined DNA methylation within the CD147/*BSG* promoter and the *BSG* gene region in cohort 2. The CD147/*BSG* gene promoter did not show differential DNA methylation patterns between non-tumor and ccRCC tissue. Methylation levels in the promoter were low and not variable in non-tumor as well as in ccRCC tissue (Figure 2C). Single CpG sites within the *BSG* gene region, i.e. cg03233876, cg23919549 and cg19651003, were differentially methylated in non-tumor and ccRCC tissue, but were not considerably associated with mRNA levels (r_S_ < 0.3; data not shown).

### CD147 mRNA levels protein expression and CD147/*BSG* promoter DNA methylation in cohort 3

CD147 mRNA levels and protein expression were examined in a third independent cohort (cohort 3) for further evaluation of the prognostic potential of CD147 for ccRCC. For this cohort, fresh frozen tissue for RNA and DNA analyses was available in addition to formalin-fixed paraffin-embedded (FFPE) tissue, which was used for the construction of TMAs to evaluate CD147 protein expression using Tissue Studio. CD147 mRNA levels were examined by TaqMan quantitative real-time PCR and were normalized to Δ-actin mRNA levels.

**Figure 3:**
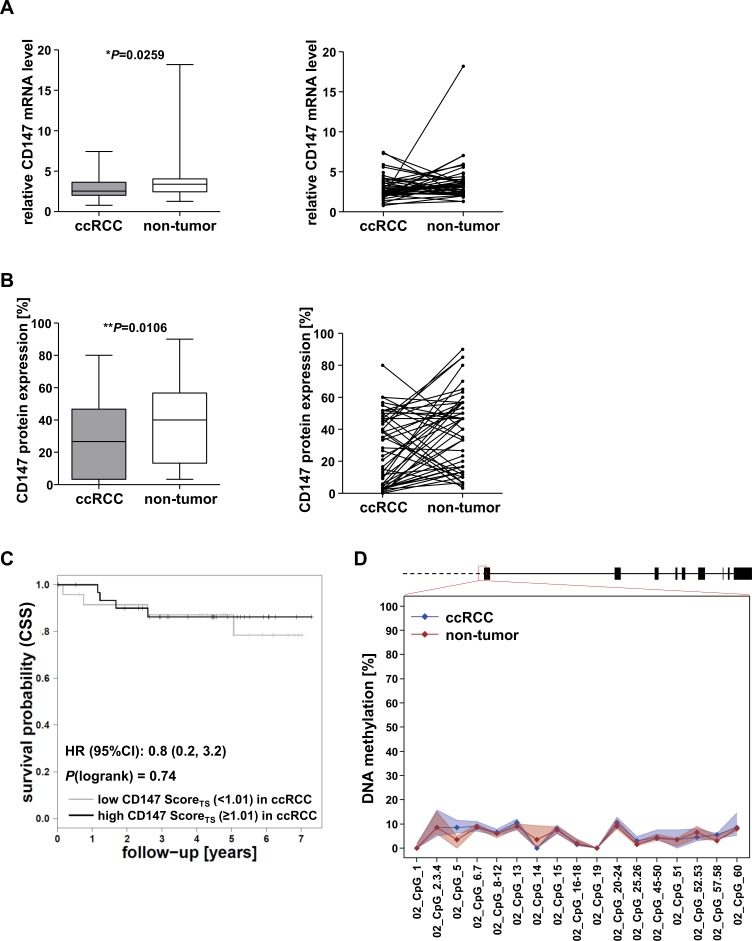
Evaluation of CD147 expression and CD147/*BSG* promoter DNA methylation in cohort 3 **A.** Relative CD147 mRNA levels in cohort 3 normalized to Δ-actin mRNA and comparison of mRNA expression levels in matching ccRCC and non-tumor samples. **B.** CD147 protein expression levels in ccRCC and non-tumor tissue and comparison in corresponding tissue samples. IHC staining was evaluated by assigning the manual composite score. **C.** The association of CD147 protein expression with cancer-specific survival (CSS) could not be validated in cohort 3. **D.** Scheme of the *BSG* gene locus and the investigated promoter region; DNA methylation levels were low in both ccRCC (blue) and non-tumor tissue (red).

On mRNA as well as on protein level, a slight decrease in expression in ccRCC compared to non-tumor tissue was observed in 64% of the cases, whereas in 36% the expression of CD147 was lower in non-tumor tissue (Figure 3A+3B). The association of CD147 protein expression with clinicopathological features (data not shown) and patient cancer-specific survival (Figure 3C) could not be confirmed in cohort 3. DNA methylation in the CD147/*BSG* 5′ gene promoter in cohort 3 was investigated by a newly established MALDI-TOF MS assay in a region identified by Kong et al, which has been shown to be important for CD147 transcription and which has already been shown to be differentially expressed in hepatocellular carcinoma (HCC) compared to normal liver tissue due to aberrant DNA methylation [15]. However, this region was invariably unmethylated in ccRCC tissue just as in corresponding non-tumor tissue (Figure 3D). These findings are in accordance to DNA methylation data in the CD147/*BSG* promoter region of the ccRCC cohort 2 (TCGA) (Figure 2C).

### Evaluation of prognostic models for ccRCC outcome

The prognostic power of CD147 expression for ccRCC outcome (cancer-specific survival) was evaluated by performing univariate Cox regression analyses and by calculating Harrell's c-indices. The results were compared to the previously identified prognostic relevance of MCT4 expression and DNA methylation at 05_CpG_8.9 in the 5′ regulatory promoter region of *SLC16A3* [8]. As shown in Table 2, CD147 protein expression was able to significantly predict patient survival only in cohort 1 (HR = 1.98, 95%CI (1.29, 3.02); *P*(logrank) = 0.0013; Harrell's c-index: 68.9%). However, the prognostic ability of MCT4 protein expression in cohort 1 was slightly better (HR = 1.03, 95%CI (1.01, 1.04); *P*(logrank) = 0.00004; Harrell's c-index: 71.9%). In the ccRCC cohort 2 (TCGA) only MCT4, but not CD147 mRNA levels and, even superior, *SLC16A3* DNA methylation in the promoter region at cg18345635 significantly predicted survival (HR = 1.60, 95%CI (1.21, 2.12); *P*(logrank) = 0.0024; Harrell's c-index: 61.4 and HR = 0.018, 95%CI (0.003, 0.119); *P*(logrank) = 0.00003; Harrell's c-index: 64.9%, respectively). Also in cohort 3, DNA methylation at the previously identified CpG site 05_CpG_8.9 was the best predictor for cancer-specific survival (HR = 0.0068, 95%CI (0.0002, 0.3022); *P*(logrank) = 0.0076; Harrell's c-index: 80.0%).

**Table 2:** Univariate Cox regression analysis of potentially prognostic factors for cancer-specific survival. The prognostic power of individual factors was estimated by calculating Harrell's c-indices

		HR (95% CI)	*P*-value (logrank)	Harrell‘s c-index
**Cohort 1**	CD147 protein expression	1.98(1.29-3.02)	0.0013	68.9%
	MCT4 protein expression	1.03(1.01-1.04)	0.00004	71.9%
**Cohort 2** (TCGA)	CD147 mRNA levels	1.08(0.83-1.40)	0.5540	52.2%
	MCT4 mRNA levels	1.60(1.21-2.12)	0.0024	61.4%
	DNA methylation at cg18345635	0.018(0.003-0.119)	0.00003	64.9%
**Cohort 3**	CD147 protein expression	0.83(0.23-2.96)	0.7780	53.7%
	MCT4 protein expression	1.03(1.00-1.06)	0.0636	70.8%
	DNA methylation at 05_CpG_8.9	0.0068(0.0002-0.3022)	0.0076	80.0%

**Figure 4:**
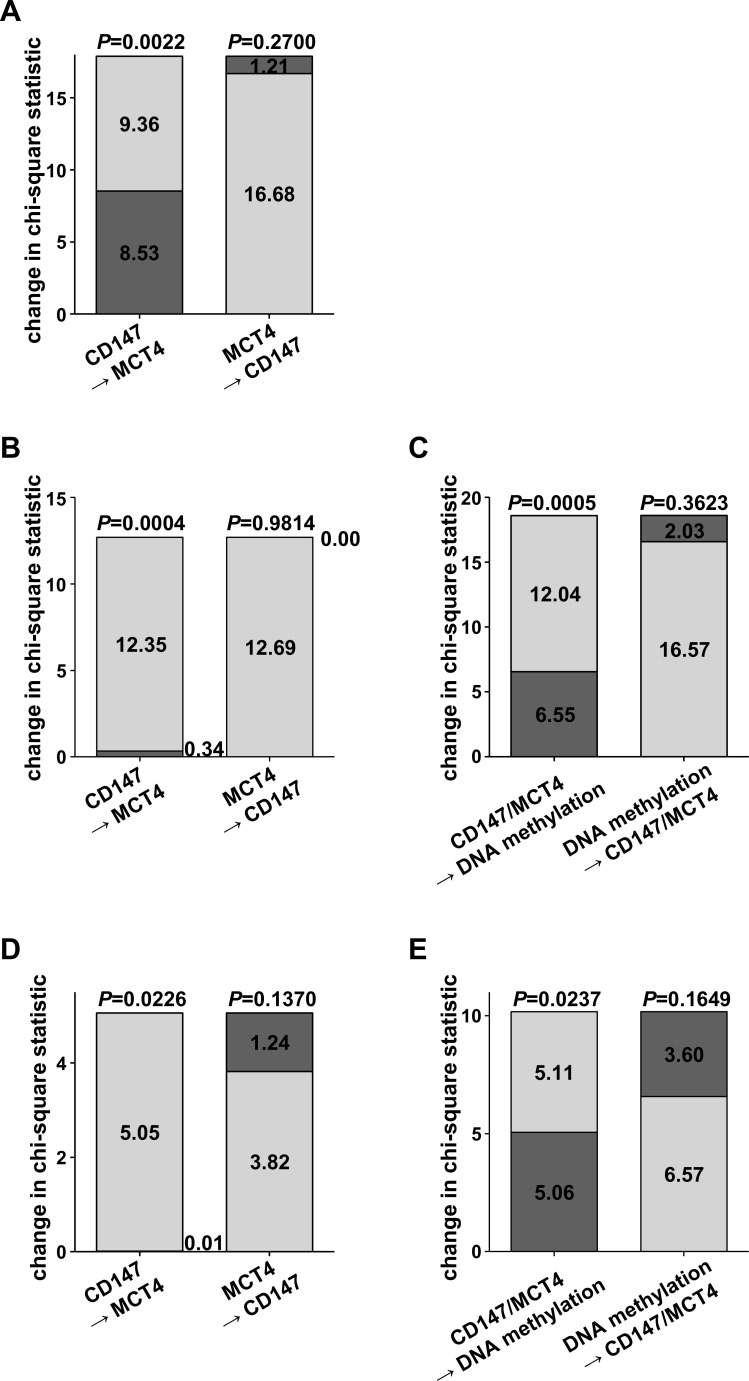
Chi-square statistics to compare Cox models comprising individual potentially prognostic parameters with Cox models derived from combinations of prognostic variables Individual contribution of **A.** CD147 and MCT4 protein expression to prognostic models in cohort 1 **B.** CD147 and MCT4 mRNA levels and **C.** DNA methylation in the *SLC16A3* promoter at cg18345635 in the ccRCC cohort 2 (TCGA) **D.** CD147 and MCT4 protein expression and **E.** DNA methylation in the *SLC16A3* promoter region at 05_CpG_8.9 in cohort 3.

In addition, Cox models for cancer-specific survival, each comprising only one of the prognostic parameters, were compared to models incorporating several variables (CD147 mRNA or protein expression, MCT4 mRNA or protein expression, DNA methylation) by computing chi-square statistics (Figure 4). In cohort 1, a model including only CD147 protein expression could be significantly improved by adding information on MCT4 protein expression. On the other hand, regarding a model comprising MCT4 protein expression only, the contribution of CD147 protein expression did not significantly increase the prognostic power (Figure 4A). Based on the TCGA data set, addition of MCT4 mRNA levels to CD147 mRNA levels resulted in a significant change in chi-square statistics, but not vice versa. However, DNA methylation at cg18345635 significantly improved the Cox model (Figure 4B+4C). This finding could be validated in cohort 3. On protein level, MCT4 contributed significantly to the prognostic model with CD147. DNA methylation at the previously identified CpG site 05_CpG_8.9 in the 5′ regulatory promoter region of MCT4/*SLC16A3* however, represented the parameter with superior prognostic relevance (Figure 4D+4E). Hazard ratios as well as confidence intervals for multivariate analyses in cohort 3 are given in Supplementary Table 1. Additional multivariate models including for instance tumor stage or the prognostic SSIGN-score, which is based on clinicopathological parameters [16], showed that neither CD147 expression nor MCT4 DNA methylation were independent prognostic factors. However, as shown previously [8], MCT4 DNA methylation at the CpG site 05_CpG_8.9 was significantly associated with clinicopathological parameters in ccRCC (data not shown).

## DISCUSSION

In this study, we examined CD147 expression, its utility as target for therapeutic intervention and its potential to predict patient outcome in ccRCC. CD147 represents the obligatory ancillary protein for correct targeting and functional expression of the monocarboxylate transporters (MCT) 1 and 4 at the plasma membrane. The prognostic ability of MCT4 or the DNA methylation in the *SLC16A3* promoter has been identified in ccRCC previously, but data for the prognostic potential of CD147 and its epigenetic regulation are limited [11, 13].

In order to guarantee accurate and reproducible quantification of CD147 protein expression in ccRCC tissue based on immunohistochemical staining of TMAs from two ccRCC cohorts, we firstly evaluated the performance of the software Tissue Studio v.3.6 (Definiens AG). We could show that Tissue Studio v.3.6 represents a feasible tool to quantify membranous biomarker expression in ccRCC tissue. Comparison of the manually assigned semi-quantitative score and the score derived from software-supported quantification of immunoreactivity revealed a strong correlation. As shown in Figure 1 few outliers with lower correlation were present. Detailed investigation of the outliers indicated that the respective cores mainly consisted of cores with < 5% tumor cells or heterogeneous tumor tissue. Although tumor tissue was evaluated histologically for TMA construction, tumor content and heterogeneity may vary depending on TMA section, indicating that quality of the tissue cores is of major importance for automated scoring of protein expression. One limitation of the automated software-based scoring remains that depending on the initial training step, certain cell types (e.g. tumor cells and cells of the tumor stroma) might be difficult to distinguish. Although we found good correlation of manual and software supported semi-quantitative scoring for the membranous marker CD147, further studies using additional biomarkers are warranted to prove reliability of software-based automated scoring for ccRCC.

The evaluation of CD147 protein expression was performed in two independent ccRCC cohorts (cohort 1 and 3) indicating substantial interindividual variability of expression. Similar variability was observed for CD147 mRNA levels in tumor tissue in the TCGA cohort as well as in our second cohort (cohort 3).

We next investigated whether this interindividual variability of expression could be explained by epigenetic regulation. Kong et al recently showed that hypomethylation in a specific CD147/*BSG* promoter region, comprising SP1 binding sites, leads to upregulation of CD147 expression and is associated with poor prognosis in HCC [15]. Liang et al also showed that CD147 hypomethylation is associated with overexpression and tumor progression in prostate cancer [17].

In ccRCC tissue, we could not find an influence of DNA methylation in the CD147/*BSG* promoter on gene expression, neither in the ccRCC cohort 2 (TCGA), nor in our cohort 3. Generally, the DNA methylation pattern of the CD147/*BSG* gene promoter was low and not variable in tumor tissue, underpinning a minor impact of DNA methylation on variability of expression in ccRCC. Instead of DNA methylation, other mechanisms, e.g. aberrant transcriptional regulation through variable expression of transcription factors, might account for interindividual variability of CD147 expression. Indeed, it has been shown that the transcription factors Sp1 and HIF1α promote CD147 expression in solid tumors [18].

Regarding the prognostic ability of CD147, univariate Cox regression analyses revealed that CD147 expression is not associated with cancer-specific survival in all of the investigated cohorts. Only in cohort 1, a significant association of CD147 protein expression and patient outcome was found. CD147 mRNA levels were not associated with cancer-specific survival, neither in the cohort 2 (TCGA) nor in our second cohort. In contrast, MCT4 expression and especially *SLC16A3* promoter DNA methylation, showed high prognostic relevance irrespective of the investigated cohort.

Comparison of prognostic models comprising individual prognostic variables or a combination of prognostic variables, revealed major contribution only for DNA methylation at the previously identified CpG site 05_CpG_8.9 in the *SLC16A3* promoter. The quantitative analysis of DNA methylation by MALDI-TOF MS represents a precise and reproducible technology ensuring reliable detection of aberrant DNA methylation levels at specific CpG sites, which is an essential requirement for the use as prognostic marker for patient outcome. In addition, the method is also suitable for the use of DNA derived from formalin-fixed paraffin-embedded (FFPE) tissue, implicating utility for the application in the clinical setting.

A recent study by Kim et al. [11] investigated expression of CD147, MCT1 and MCT4 in ccRCC based on immunohistochemical staining of TMAs and analyses of RNAseq data of the ccRCC cohort of TCGA. They showed that in their cohort not MCT4, but CD147 and MCT1 were predictive for disease progression. Several reasons may explain the different results compared to our study. We used a different primary antibody for staining of MCT4 in our study, which was validated by siRNA experiments [8]. Most importantly, different outcome endpoints were used for data analysis. Based on RNAseq data of the ccRCC cohort of TCGA, they did not find an association of MCT4 expression with overall survival. In our study, we defined cancer-specific survival as recently proposed by Gulati et al. [19] as the relevant endpoint in biomarker studies for prediction of patient outcome in ccRCC. Additionally, we used latest follow-up data of the ccRCC cohort of TCGA (downloaded in May 2015). Of note, with cutoff points determined by conditional inference tree models, we even found a significant association of MCT4 mRNA expression and overall survival for ccRCC patients in the TCGA cohort, which was however weaker than the association with cancer-specific survival.

Though CD147 might play pivotal roles in carcinogenesis and is considerably expressed in ccRCC tissue, a major limitation for the use of CD147 as therapeutic target is that it is also expressed in non-tumor tissue and fulfills also vital physiological functions there. Therapeutic intervention would therefore also harm healthy renal tissue. MCT4 protein however is expressed at significantly higher levels in ccRCC compared to non-tumor tissue and therefore represents the better candidate also for therapeutic intervention. MCT4 specific inhibitors are currently under development by Astra Zeneca and are expected to inhibit tumor growth [20]. Alternatively, the aberrant *SLC16A3* promoter DNA methylation could be exploited for therapeutic intervention. An attractive strategy would be targeted DNA re-methylation to silence MCT4 expression. However, further studies are required to fully evaluate the potential of “gene editing” using, e.g., zinc finger, TALEN or CRISPR/Cas9 technologies to modify DNA methylation at selected CpG sites.

In summary, our study suggests that the contribution of CD147 protein expression to the prediction of cancer-specific survival in ccRCC is limited. The prognostic significance was outperformed by the prognostic power of MCT4 expression and especially by that of DNA methylation at specific CpG sites in the *SLC16A3* promoter region, corroborating the role of MCT4 as qualified prognostic biomarker for ccRCC.

## MATERIALS AND METHODS

### Patient samples

Two independent retrospective cohorts of ccRCC patients (cohort 1 and cohort 3) treated at the Department of Urology of the University of Tuebingen, Tuebingen, Germany, were investigated in this study. Cohort 1 comprises 186 ccRCC patients of Caucasian origin who underwent partial or radical nephrectomy between 1993 and 2007. Formalin-fixed paraffin-embedded (FFPE) ccRCC and tumor-surrounding normal tissue as well as detailed documentation of clincopathological parameters were available for each patient. The second independent ccRCC cohort (cohort 3) consists of 59 consecutive patients, diagnosed and treated between 2007 and 2010. In addition to FFPE tissue, surgically resected specimens of fresh frozen ccRCC and adjacent non-tumor tissue were available for this cohort. Detailed patient characteristics, clinicopathological features and survival data are given in Table 1. Informed written consent was provided by each subject prior to surgical resection and the use of the tissue was approved by the ethics committee of the University of Tuebingen. Further details about sample collection and definition of clinical endpoints of the two cohorts are explained in Supplementary Material. In addition, a third independent cohort of ccRCC patients from The Cancer Genome Atlas (TCGA) was investigated (cohort 2). Publically available open access clinical data, RNAseq data (*n* = 529 for ccRCC, *n* = 72 for non-tumor, *n* = 510 with information about cancer-specific survival (CSS)) and DNA methylation data (Illumina Infinium HumanMethylation 450K BeadChip; *n* = 315 for ccRCC, *n* = 160 for non-tumor, *n* = 303 with information about CSS) were used for the analyses. Characteristics of cohort 2 from TCGA are summarized in Table 1. Processed data sets were obtained from the TCGA Data Portal (http://tcga-data.nci.nih.gov/tcga/) using the Data Browser tool. For further details see Supplementary Material.

### CD147 immunohistochemical staining and TMA analysis

#### Slide preparation

For the investigation of CD147 protein expression, tissue microarrays (TMAs) containing ccRCC and corresponding non-tumor tissue of the cohorts 1 and 3 were constructed. The TMA slides were processed as previously described [21] and immunostained as described in [8] using an antibody against CD147 (1:5000; Abcam, Cambridge, UK). The antibody was validated by CD147 siRNA knockdown in four RCC cell lines (Supplementary Figure S1). CD147 stained TMA slides were scanned using the slide scanner SCN400 (Leica microsystems, Wetzlar, Germany).

#### Semi-quantitative scoring

For manual evaluation of CD147 protein expression in non-tumor and ccRCC tissue a semi-quantitative score derived from staining intensity and the percentage of stained cells was applied by two independent investigators blinded to patient's clinical characteristics. Staining intensity was graded into negative (0), weak (1), medium (2) and strong (3). The composite IHC score was obtained by multiplication of the staining intensity with the percentage of stained cells (0-100%). These immunoreactivity score values of 0-300 were finally converted to percentage of protein expression (0-100%).

#### Automated scoring

For automated evaluation of CD147 protein expression the software-based image analysis software Tissue Studio v.3.6 (Definiens AG, Munich, Germany) was applied. The predefined analysis solution ‘Nuclei, Membranes and Cells’ with the tasks ROI detection, nucleus and membrane detection, and cell classification was used. Regions of interest were determined to discriminate between tumor tissue (either IHC positive or negative) and e.g. fibrotic tissue or regions of hemorrhage. Only tumor regions were used for further evaluation. Threshold settings for nucleus and membrane detection were adjusted. Detected cells were subclassified as negative, low, medium or high according to IHC staining intensity. The Tissue Studio score (Score_TS_) was derived from the number of cells in tumor regions (IHC positive and negative), multiplied by their staining intensity and divided by the number of all detected cells in tumor regions:

### RNA isolation and quantification

Total RNA was isolated from fresh frozen tissue of cohort 3 using the mirVana^TM^ miRNA Isolation Kit (ambion, life technologies, Darmstadt, Germany) according to manufacturer's protocol. RNA integrity was analysed using Agilent RNA 6000 Nano Kit and the Agilent Bioanalyzer (Agilent technologies, Waldbronn, Germany). Only high quality RNA was used for further investigations. For subsequent cDNA syntheses, the High capacity cDNA reverse transcription Kit (with RNAse inhibitor) (Applied Biosystems, Foster City, USA) was used according to manufacturer's instructions. Relative CD147 mRNA quantity was examined by TaqMan quantitative real-time PCR with a predeveloped TaqMan gene expression assay (Applied Biosystems, Foster City, USA) using the BioMark system (Fluidigm, South San Francisco, CA, USA). As previously described [8], reverse transcribed cDNA was preamplified in 14 cycles using the TaqMan PreAmp Master Mix Kit (Applied Biosystems, Foster City, USA), diluted 1:5 in DNA suspension buffer and analysed in triplicates using the BioMark Instrument using the BioMark Gene Expression Data Analysis software. CD147 mRNA levels were normalized to the mRNA expression of β-actin.

### DNA methylation analyses

Genomic DNA was isolated from fresh frozen tissue samples of cohort 3 using the QIAmp DNA mini Kit (Qiagen, Hilden, Germany) according to manufacturer's instructions. Bisulfite treatment was performed with the EZ DNA methylation Gold^TM^ Kit (Zymo Research, Irvine, USA) as stated by the manufacturer. Quantitative analyses of DNA methylation in the *BSG* promoter were performed by matrix-assisted laser desorption ionization time of flight mass spectrometry (MALDI-TOF MS) as described previously. Amplicons were generated, which cover the promoter region important for *BSG* transcription [15]. Sequence information was retrieved from GenBank database (accession number: NC_000019.10, GeneID: 682). The software Methprimer was used for the design of primers for methylation-specific PCR amplification (http://www.urogene.org/methprimer/). Forward primers included a 10-mer tag and a T7 polymerase promoter tag was attached to reverse primer sequences to enable subsequent reverse transcription using the MassCLEAVE reagent Kit (Sequenom, San Diego, USA). The single stranded RNA strands generated by reverse transcription were cleaved base specifically by RNase A, in parallel and subsequently purified using clean resin (Sequenom, San Diego, USA). Samples were spotted on spectroCHIP arrays using the MassARRAY nanodispenser. Mass spectra were obtained using the MassARRAY compact system and evaluation of spectra methylation ratios was performed using the EpiTYPER 1.0 software.

$${\text{Score}}_{\text{TS}}=\frac{\text{IHCneg}(\#\text{neg}\times 0+\#\text{low}\times 1+\#\text{med}\times 2+\#\text{high}\times 3)+\text{IHCpos}(\#\text{neg}\times 0+\#\text{low}\times 1+\#\text{med}\times 2+\#\text{high}\times 3)}{\#\text{IHCneg}+\#\text{IHCpos}}$$

### Statistical analyses

Statistical analyses were performed with R-3.1.2 (http://www.r-project.org) [22] including additional packages boot_1.3-15 [23], party_1.0-20 [24], and survival_2.37-7 [25] as well as GraphPad Prism 5.0 (GraphPad Software, Inc.). Statistical significance was defined as *P* < 0.05.

Correlation analyses between the manual CD147 protein expression score and the CD147 protein expression score derived from Tissue Studio v.3.6 were performed by Spearman correlation tests. Differences in values of CD147 mRNA levels and protein expression between ccRCC and non-tumor tissue were investigated by Wilcoxon-Mann-Whitney tests.

Cox proportional hazard regression models were used to study the association between cancer-specific survival (CSS) and DNA methylation, mRNA levels, and protein expression as well as clinicopathological parameters or the prognostic SSIGN-score. Corresponding Harrell's c-indices were calculated based on 1000 bootstrap replicates.

Comparisons of Cox models were performed by analysis of deviance. Conditional inference trees were applied to define mRNA or protein expression cutoffs for prediction of CSS. For resulting cutoffs, Kaplan-Meier estimates along with hazard ratios and log-rank tests were computed.

## SUPPLEMENTARY MATERIAL FIGURES AND TABLE


