# Supplementary material for: MCT4 surpasses the prognostic relevance of the ancillary protein CD147 in clear cell renal cell carcinoma
Source: Oncotarget. 2015 Sep 10;6(31):30615–27. doi: 10.18632/oncotarget.5593 (PMC4741556; doi:10.18632/oncotarget.5593)
Supplement: Supplementary file 1 [file oncotarget-06-30615-s001.pdf]

MCT4 surpasses the prognostic relevance of the ancillary protein CD147 in clear cell renal cell carcinoma

Supplementary Material

Supplementary Methods

Patient Cohort

The patients of cohort 1 and 3 were treated at the Department of Urology, University Hospital Tuebingen, Tuebingen, Germany. Samples were obtained from representative tumor regions and adjacent tumor surrounding non-tumor tissue. Surgically resected ccRCC tissues were classified according to the seventh edition of the Union Internationale Contre le Cancer/American Joint Committee on Cancer system (2009). Pathologists assured that the investigated samples derived from tumor surrounding normal tissue were histologically unsuspicious and not invaded by the tumor.

The follow-up process included examination of patients by axial imaging at the time of surgery, postoperatively every three to four months during the first year and semiannually in the second and third year of follow-up. Annual clinical assessment consisted of chest x-ray or thoracic computerized tomography, abdominal sonography, computerized tomography or magnetic resonance imaging, and serum chemistry. Survival end-point was cancer-specific survival (CSS) defined as the time from surgery to death or censored if alive at the last date of follow-up until November 2014. Data for patients who died from other causes than ccRCC disease were censored at the time of death. None of the patients in the cohort 1 and cohort 3 received any kind of neoadjuvant therapy before surgery, neither immune- nor chemotherapy.

Expression (Illumina HiSeq2000; TCGA_KIRC_exp_HiSeqV2-2015-02-24) and DNA methylation (Illumina Infinium HumanMethylation 450K BeadChip; TCGA_KIRC_hMethyl450-2015-02-24) data sets of the ccRCC samples (cohort 2) collected by The Cancer Genome Atlas (TCGA) were downloaded from the Cancer Browser (https://genome-cancer.soe.ucsc.edu) on May 15, 2015. Both data sets also included corresponding follow-up data. In addition, clinical and follow-up data of the ccRCC cohort 2 were assessed directly from TCGA data portal (https://tcga-

data.nci.nih.gov/tcga; nationwidechildrens.org_clinical_follow_up_v1.0_kirc.txt and nationwidechildrens.org_clinical_patient_kirc.txt; Version 2.0.25.0May 2015). For n=530 primary ccRCCs and n=208 non-tumor samples, clinical data from TCGA portal as well as expression data and/or methylation data was available. Thereof, expression data was accessible for n=529 primary ccRCCs and n=72 non-tumor samples and methylation data for n=315 primary ccRCCs and n=160 non-tumor samples.

CSS was defined according to Gulati et al (1), in which always the longest follow-up of all data sources described above was considered. In case of conflicts, CSS was set to NA (not available), therefore leaving 510 and 303 primary ccRCCs for survival analyses of expression and DNA methylation data, respectively. Moreover, consistent CSS, expression, as well as DNA methylation data was available for 302 patients.

CD147 knockdown

For the validation of the CD147 antibody, CD147 was silenced by siRNA mediated knockdown in the renal cell carcinoma cell lines A498, 786-O, Caki1 and Caki2 (CLS, Eppelheim, Germany). A498 and 786-O cells were grown in EMEM or DMEM/Ham's F12, respectively, supplemented with 2 mM L-glutamine and 10% fetal bovine serum. Caki1 and Caki2 cells were grown in McCoy's 5A with 10% fetal bovine serum and 50 µg/ml gentamicin. CD147 knockdown was performed using a pool of CD147-targeting siRNAs (siGENOME SMARTpool – human BSG, Thermo Scientific, Lafayette, USA). Untreated cells and cells transfected with a pool of non-targeting siRNAs (siGENOME non-targeting siRNA pool #1, Thermo Scientific, Lafayette, USA) were used as controls. Cells were seeded 24h prior to transfection. A final siRNA concentration of 25 nM was transfected using DharmaFECT® transfection reagent (Thermo Scientific, Lafayette, USA) according to manufacturer's instructions. Cells were harvested after 72h of incubation. CD147 knockdown was assessed by western blot analysis.

Western Blot

Crude membrane fractions were prepared from CD147-siRNA transfected A498, 786-O, Caki1 and Caki2 cells and respective controls as described previously (2). 10 µg protein of the membrane fraction were diluted in hypotonic buffer to a volume of

20 µl and denatured at 37°C for 30 min in Laemmli sample buffer right before separation on a 10% SDS/polyacrylamide gel by electrophoresis using the Mini-PROTEAN® Tetra system (Biorad, München, Germany). Wet transfer in cold blotting buffer was applied to blot proteins onto a nitrocellulose membrane by use of the Mini Trans-Blot® Electrophoretic Transfer Cell (Biorad, München, Germany). The membrane was blocked for 1 h with 5% (wt/vol) skim milk in TBST and subsequently incubated with primary CD147 antibody (ab78106, abcam, Cambridge, UK) diluted 1:5000 in TBST over night at 4°C. After washing with TBST, the membrane was incubated for 1 h at room temperature with a goat anti-mouse horseradish peroxidase-conjugated secondary antibody (Santa Cruz Biotechnology, Santa Cruz, USA) diluted 1:5000 in TBST. After washing with TBST, the membrane was incubated with SuperSignal® West Dura Extended Duration Substrate (Pierce, Rockford, USA) to detect horseradish peroxidase activity. Chemoluminescence was detected using the Stella 3200 Imaging System (Raytest, Straubenhardt, Germany). Stripping of the membrane was performed by 30 min incubation in Restore™ Western Blot Stripping Buffer for subsequent detection of β-actin. The primary β-actin antibody (Sigma-Aldrich, St. Louis, USA) and secondary goat anti-mouse horseradish peroxidase antibody (Santa Cruz Biotechnology, Santa Cruz, USA) were used at dilutions of 1:5000 in TBST incubated for 1 h at room temperature.

Supplementary Table 1: Multivariate Cox regression analysis of potentially prognostic factors for cancer-specific survival, including CD147 and MCT4 protein expression (cohort 1), CD147 and MCT4 mRNA as well as DNA methylation (cohort 2), CD147 and MCT4 protein expression as well as DNA methylation (cohort 3).

	**Variable**	**HR (95% CI)**
**Cohort 1**	CD147 protein expression	1.32 (0.81-2.13)
	MCT4 protein expression	1.02 (1.01-1.04)
**Cohort 2** (TCGA)	CD147 mRNA levels	0.98 (0.63-1.53)
	MCT4 mRNA levels	1.27 (0.86-1.87)
	DNA methylation at cg18345635	0.03 (0.004-0.22)
**Cohort 3**	CD147 protein expression	0.21 (0.03-1.25)
	MCT4 protein expression	1.03 (0.97-1.09)
	DNA methylation at 05_CpG_8.9	0.004 (0.0000-0.76)

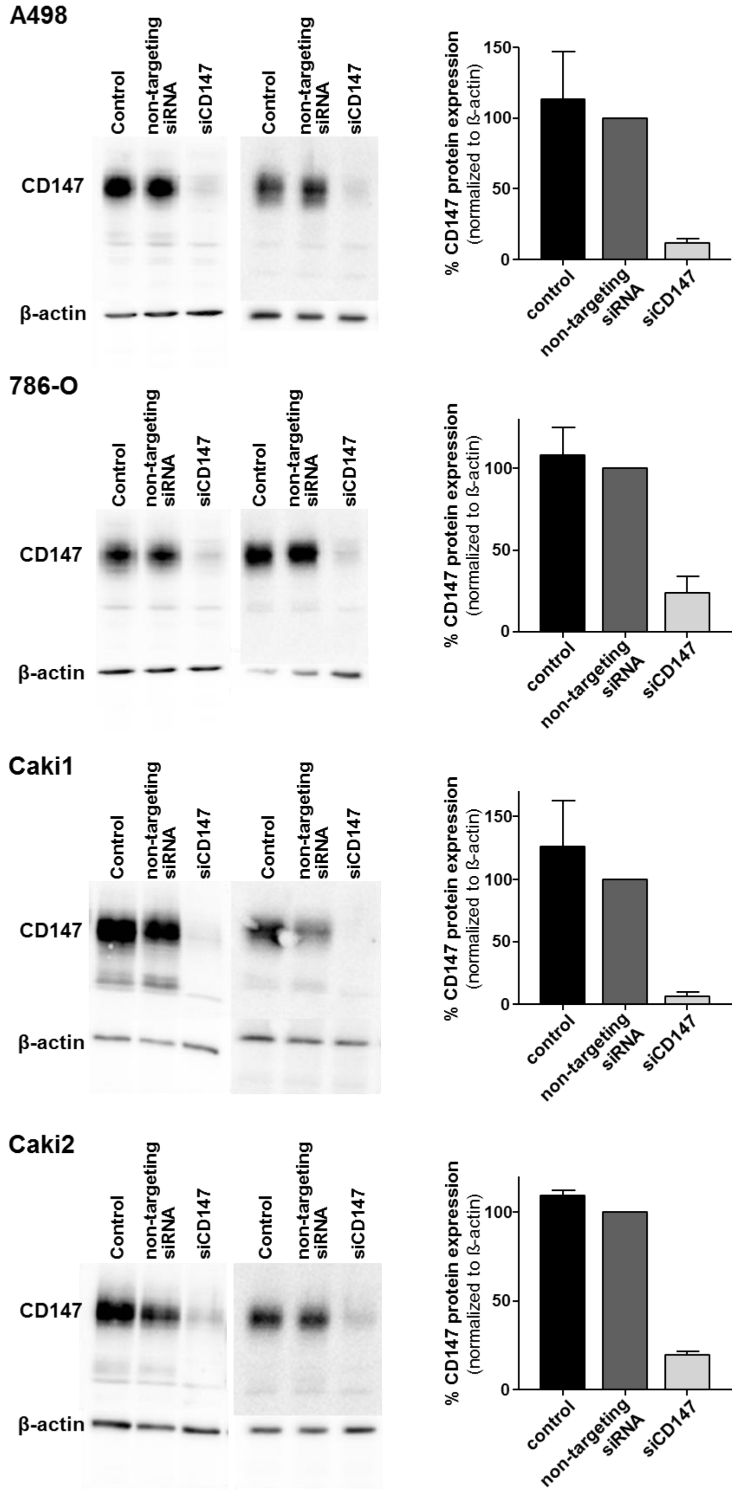

Supplementary Figure S1: Validation of the CD147 antibody by siRNA mediated knockdown of CD147 in four RCC cell lines. Representative Western Blots of two independent experiments are shown (left). Percentage of CD147 protein expression was calculated based on three independent experiments and normalized to CD147 expression in non-targeting siRNA transfectants.

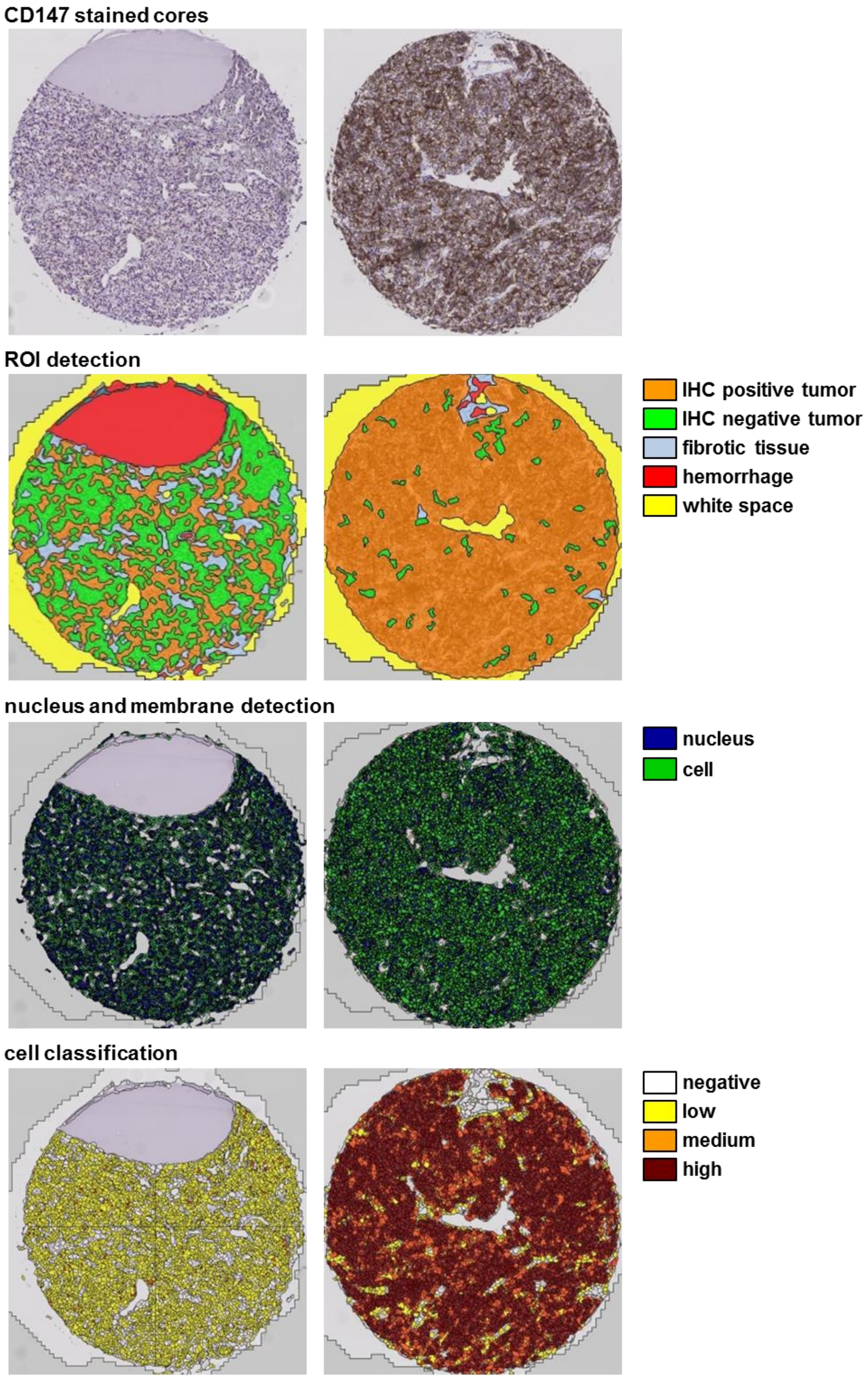
CD147 stained cores
ROI detection
IHC positive tumor
IHC negative tumor
fibrotic tissue
hemorrhage
white space
nucleus and membrane detection
nucleus
cell
cell classification
negative
low
medium
high

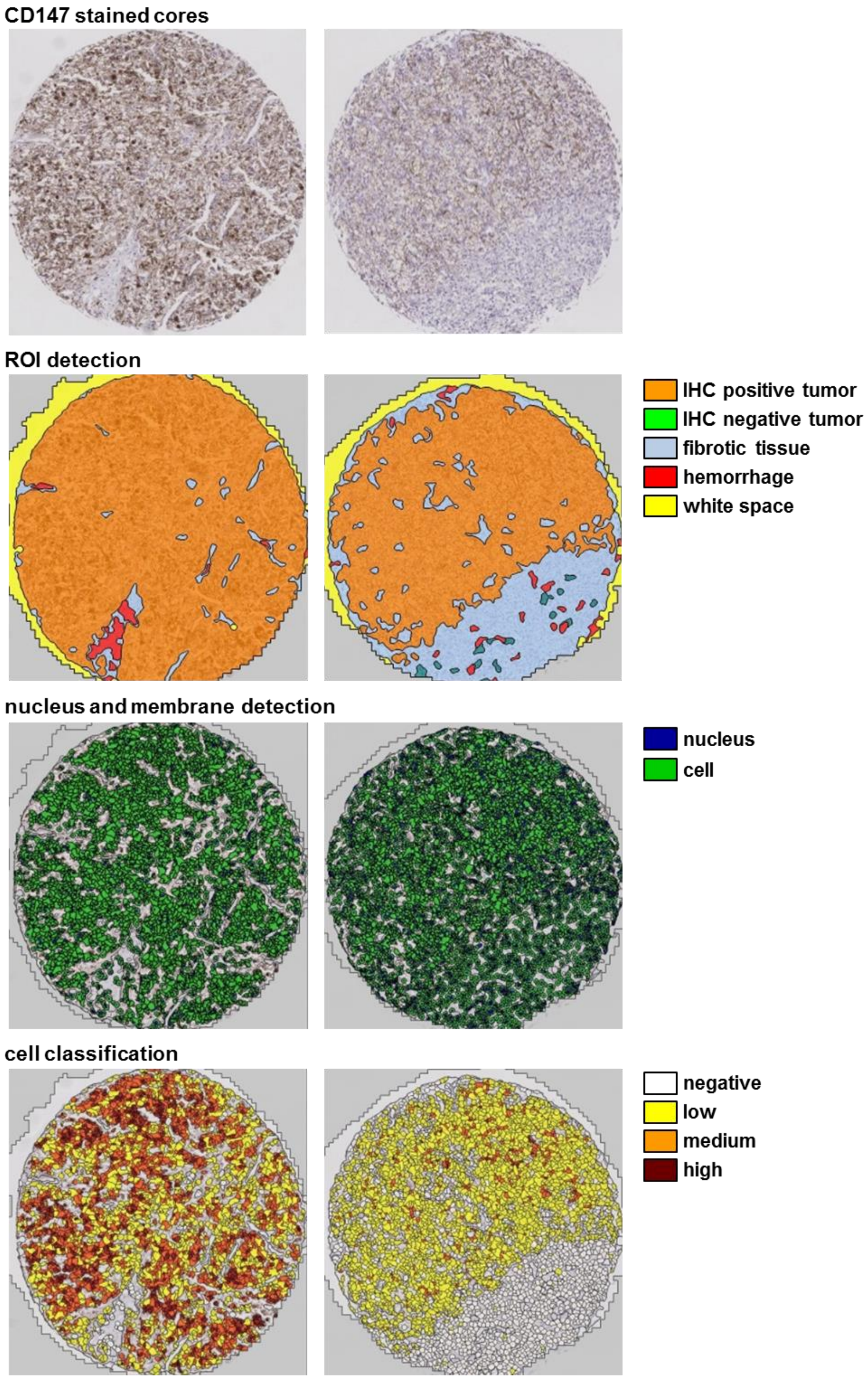

Supplementary Figure S2: Software-supported evaluation of CD147 protein expression with Tissue Studio (Definiens AG). Screenshots of the performed steps using the predefined analysis solution 'Nuclei, Membranes and Cells' with the tasks ROI detection, nucleus and membrane detection, and cell classification.

References

(1) Gulati S, Martinez P, Joshi T, Birkbak NJ, Santos CR, Rowan AJ, Pickering L, Gore M, Larkin J, Szallasi Z, Bates PA, Swanton C, Gerlinger M. Systematic evaluation of the prognostic impact and intratumour heterogeneity of clear cell renal cell carcinoma biomarkers. Eur Urol. 2014; 66: 936-48.

(2) Fisel P, Kruck S, Winter S, Bedke J, Hennenlotter J, Nies AT, Scharpf M, Fend F, Stenzl A, Schwab M, Schaeffeler E. DNA methylation of the SLC16A3 promoter regulates expression of the human lactate transporter MCT4 in renal cancer with consequences for clinical outcome. Clin Cancer Res. 2013; 19: 5170-81.
